# Case Report: Pediatric Recurrent Acute Liver Failure Caused by Neuroblastoma Amplified Sequence (*NBAS*) Gene Mutations

**DOI:** 10.3389/fped.2020.607005

**Published:** 2021-01-13

**Authors:** Bingxin Jiang, Fangfei Xiao, Xiaolu Li, Yongmei Xiao, Yizhong Wang, Ting Zhang

**Affiliations:** ^1^Department of Gastroenterology, Hepatology and Nutrition, Shanghai Children's Hospital, Shanghai Jiao Tong University, Shanghai, China; ^2^Institute of Pediatric Infection, Immunity and Critical Care Medicine, Shanghai Children's Hospital, Shanghai Jiao Tong University School of Medicine, Shanghai, China

**Keywords:** recurrent acute liver failure, whole-exome sequencing, mutational spectrum, neuroblastoma amplified sequence, compound heterozygote mutation

## Abstract

Acute liver failure (ALF) in childhood is a rapidly progressive, potentially life-threatening condition that occurs in previously healthy children of all ages. However, the etiology of ~50% of cases with pediatric ALF remains unknown. We herein report a 4-year-old Chinese girl with recurrent ALF (RALF) due to a mutation in the neuroblastoma amplified sequence (*NBAS*) gene. The patient had suffered from multiple episodes of fever-related ALF since early childhood. She had also suffered from acute kidney injury, hypertension, mild pulmonary hypertension, pleural effusion, and hypothyroidism. A novel compound heterozygote mutation, c.3596G> A (p.C1199Y)/ex.9del (p.216-248del), in the *NBAS* gene was identified by whole-exome sequencing (WES). The missense mutation c.3596G> A (p. C1199Y) was inherited from her father, and ex.9del (p.216-248del) was inherited from her mother. The patient was managed with intensive treatments, such as renal replacement therapy (CRRT), intravenous antibiotics, and glucose infusion, and was discharged after full recovery. We identified a novel compound heterozygote mutation in the *NBAS* gene that caused fever-related RALF in a Chinese child, which further expands the mutational spectrum of *NBAS*.

## Introduction

Acute liver failure (ALF) in childhood is a rapidly progressive, potentially life-threatening condition that occurs in previously healthy children of all ages. Multiple etiologies have been identified as the causes of ALF in childhood, such as infection, inborn errors of metabolism, drug poisoning, abnormal perfusion, and autoimmune diseases. However, the etiologies of ~50% of cases with pediatric ALF remain unknown (1). Recently, neuroblastoma amplified sequence (*NBAS*) gene mutations were identified as a novel cause of pediatric recurrent acute liver failure (RALF) by Haack et al. (2). The *NBAS* gene is located on chromosome 2p24.3 and has 52 exons encoding a protein containing 2,371 amino acids (NCBI GENE ID: 51594). Due to its wide expression, *NBAS* gene mutations can induce a wide range of clinical symptoms, ranging from isolated fever-related RALF

to a multisystemic phenotype including short stature, skeletal defects, retinal dystrophy and optic atrophy, status epilepticus, immune dysregulation, and immunodeficiency (3). Here, we report a child with RALF caused by a novel compound heterozygous mutation in *NBAS*. The clinical features and genetic variants of the patient were described in this study.

## Case Presentation

A 4-year-old girl with a past history of RALF was admitted to our hospital due to nausea and vomiting with a high fever (39.5°C). She had no symptoms of abdominal pain and distention or diarrhea, no chills or convulsions, and no headache, dizziness, diplopia, or blurred vision. The girl was born at full-term and met normal developmental milestones. There was no family history of hereditary or metabolic liver diseases. Physical examination (PE) of the patient showed normal cognitive function without confusion, personality changes, disorientation, or consciousness disturbance. It also revealed mild jaundice, enlarged tonsils, and palpable hepatomegaly (1–2 cm below the costal margin) without splenomegaly. Nervous system examination revealed normal muscle tone and strength, a normal Babinski sign, and negativity for ankle clonus. Laboratory data showed abnormal liver function [direct bilirubin (DB) 24.50 μmol/L (normal range: 0–6.8), total bilirubin (TB) 35.81 μmol/L (normal range: 3.4–17.1), alanine transaminase (ALT) 7169 U/L (normal range: 5–40), aspartate transaminase (AST) 9677 U/L (normal range: 8–40), γ-glutamyl-transferase (γ-GGT) 40 U/L (normal range: 7–32 U/L), and albumin 14 g/L (normal range: 38–54 g/L)]. The lactate dehydrogenase [>9480 U/L (normal range: 110–290 U/L)] and blood ammonia [90 μmol/L (normal range: 11–51 μmol/L)] levels were significantly increased. Coagulopathy [prothrombin time (PT) 20.1 s (normal range: 9.8–12.1 s), international normalized ratio (INR) 1.76 (normal range: 0.82–1.25), thrombin time (TT) 22.1 s (normal range: 14–21 s)] and renal dysfunction [serum creatinine (Cre) 524 μmol/L (normal range: 18–66), blood urea nitrogen (BUN) 35.6 mmol/L (normal range: 0–8.3)] were observed. Thyroid function tests were performed, with the results supporting a diagnosis of hypothyroidism [triiodothyronine (T3) 0.81 nmol/L (normal range: 1.34–3.70), thyroxine (T4) 54.04 nmol/L (normal range: 64.30–158.70), free triiodothyronine (FT3) 2.84 pmol/L (normal range: 3.58–6.92), free thyroxine (FT4) 9.35 pmol/L (normal range: 9.60–14.50), and thyroid-stimulating hormone (TSH) 0.10 μIU/ml (normal range: 0.90–4.00)]. The detection of liver-damaging viruses, such as Epstein–Barr virus, cytomegalovirus, herpes simplex virus, hepatitis A virus, hepatitis B virus, and hepatitis C virus, was all negative. Ceruloplasmin and α1-antitrypsin were all normal. Tandem mass spectrometry (TMS) for metabolic disorder screening was unremarkable. Analyses of autoantibodies, including autoimmune hepatitis-related autoantibodies, were all negative. Mitochondrial gene analysis was carried out, but no abnormalities were observed. Abdominal ultrasound showed hepatomegaly and mild ascites but no splenomegaly. Echocardiography showed mild pulmonary hypertension. The electrocardiogram (ECG) was normal. In addition, no abnormalities were observed by brain magnetic resonance imaging (MRI).

**Figure 1:**
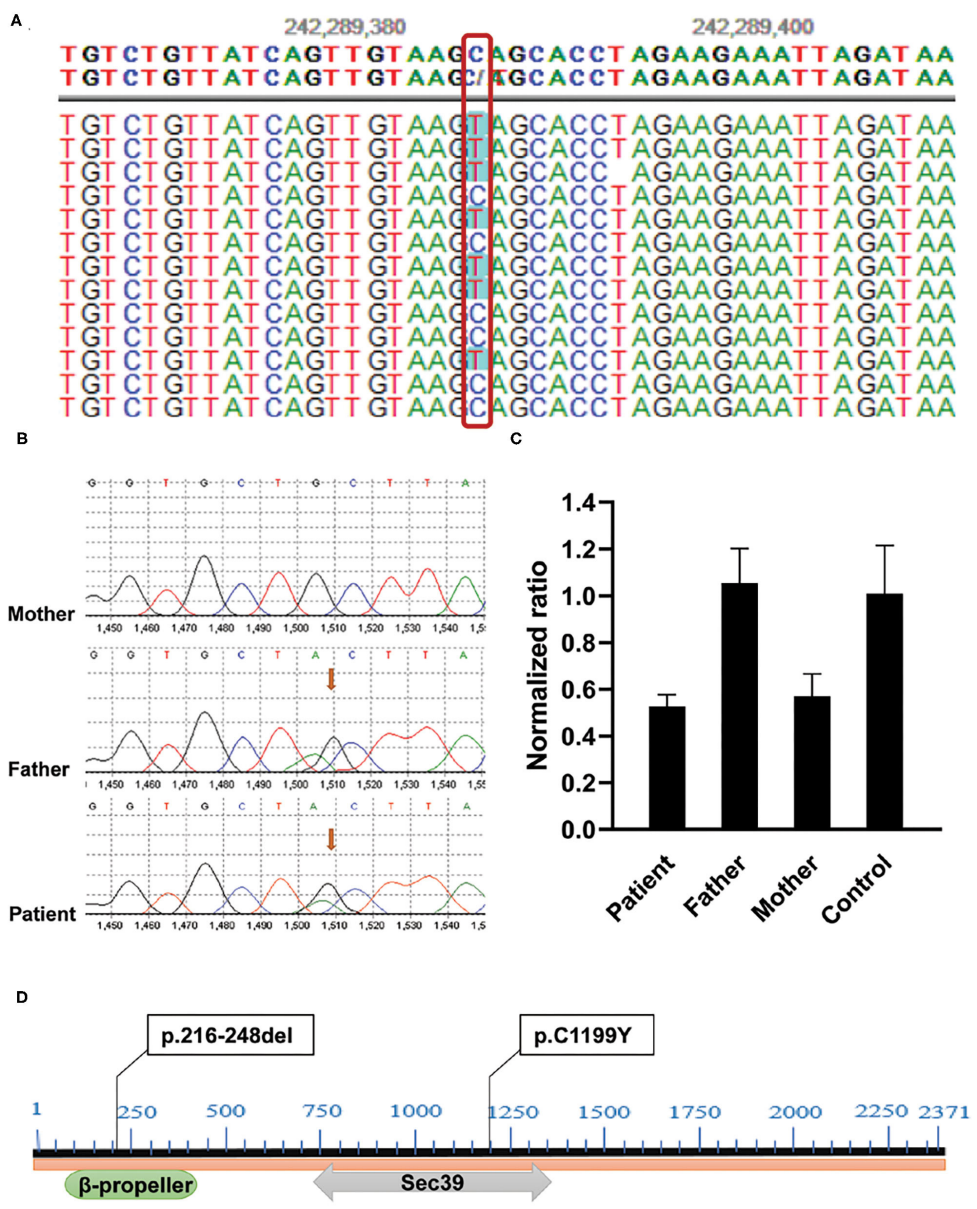
Identification of compound heterozygous mutations in the *NBAS* gene in the family. **(A)** c.3596G>A (p.C1199Y) mutation was found by WES. **(B)** Sanger sequencing showed that the mutation c.3596G> A (p.C1199Y) was inherited from her father. **(C)** Validation of ex.9del (p.217-249del) mutation by quantitative PCR. Genomic DNA was extracted from PBMCs of the patient, her parents, and healthy control. Reactions were set up using one set of primers upstream (Forward: 5'-GCTGTGTTGATTCCATGAGGA-3') and downstream (Reverse: 5'-AGTGTTGGAACAAATCAGAGCTA-3') of exon 9 in triplicate. The β-actin gene served as reference gene. The 2^−ΔΔ*CT*^ method was used to calculate the copy number. Sample with normalized ratio (NR) <0.1 denotes homozygous deletion individual, sample with NR about 0.5 denotes heterozygous deletion individual, sample with NR about 1 denotes normal individual (two copies), and sample with NR about 1.5 or more denotes copy number gain individual. **(D)** The location of two variants in NBAS protein: c.3596G>A (p.C1199Y) locates in C-terminal β-propeller region, and ex.9del (p.217-249del) mutation locates in the Sec39 domain.

Considering that no etiological diagnosis was reached despite mitochondrial gene sequencing, the patient was subjected to whole-exome sequencing (WES) using genomic DNA extracted from peripheral blood mononuclear cells (PBMCs). Exome capture was carried out using IDTxGen Exome Research Panel (IDT, USA) and paired-end sequencing was performed on HiseqX10 (Illumina, USA). Sequencing data were analyzed to identify sequence variants [single nucleotide variant (SNV), insertion/deletion (Ins/Del)] and copy number variants (CNVs) using an in-house pipeline (Fulgent genetics). A phenotype-driven gene list was created to perform a primary variant interpretation for a more targeted analysis (4). After data analysis, variant filtering, and prioritization, a novel compound heterozygote mutation, c.3596G> A (p.C1199Y)/ex.9del (p.216-248del) in *NBAS* was identified (Figure 1A). No other potentially pathogenic mutations were detected in genes predisposing to ALF (e.g., TRMU, OMIM: 613070; MARS OMIM:615486; LARS, OMIM: 615438; RINT1, OMIM: 618641) by WES of the patient. The mutations were verified by Sanger sequencing and quantitative PCR, respectively (Figures 1B,C). Genotyping of the unaffected parents showed the mutation c.3596G> A (p. C1199Y) in the father and the ex.9del (p.216-248del) mutation in the mother. The c.3596G> A (p. C1199Y) mutation has been previously reported in patients with *NBAS* deficiency (5–7), but the ex.9del (p.216-248del) mutation has not been described previously. *In silico* predictors predict that the variant c.3596G> A (p. C1199Y) is located in a highly conserved amino acid residue (Sec39 domain) that affects the structure/function of the *NBAS* protein and is defined as pathogenic (SIFT score: 0.000, http://sift.jcvi.org; PROVEAN score: −9.563, http://provean.jcvi.org/ index.php). The ex.9 del (p.216-248del) variant located in the β-propeller leads to the deletion of exon 9 and is predicted to be deleterious by PROVEAN with a score of −123.936 (Figure 1D). The two variants were classified as likely pathogenic according to the guidelines of the American College of Medical Genetics and Genomics (ACMGG).

**Figure 2:**
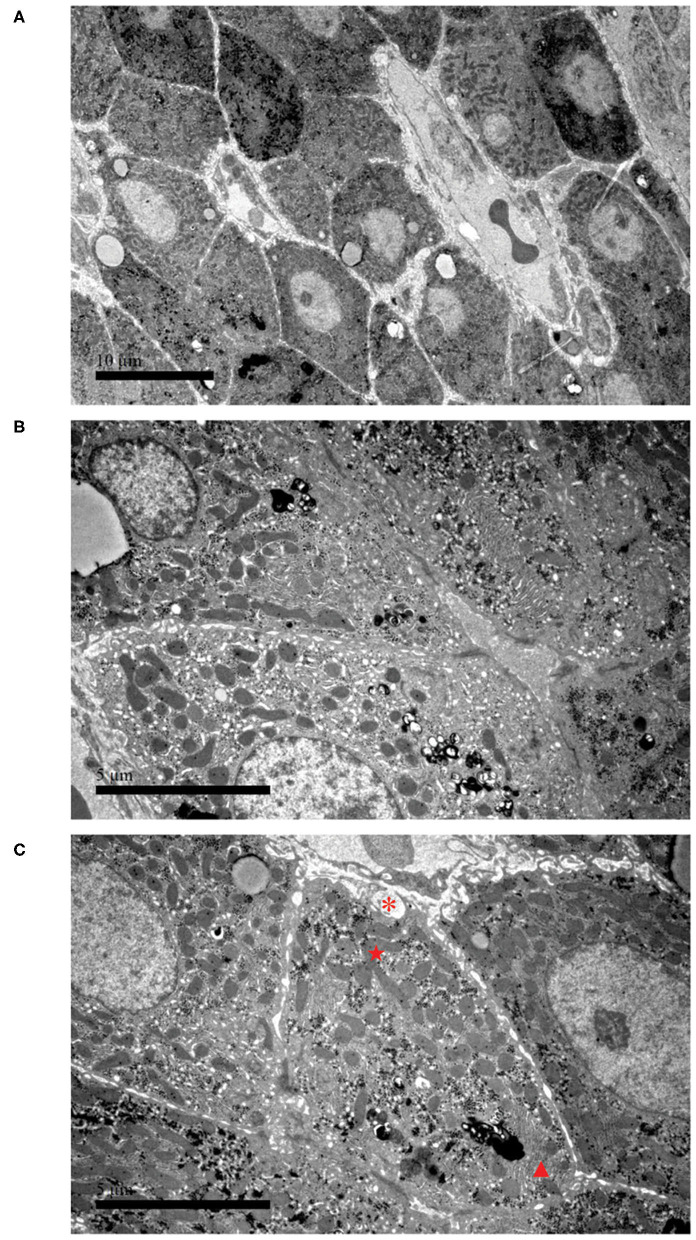
Electron microscopy of liver biopsy. **(A)** Hepatocytes are irregular in shape and size, and the nucleus is eccentric (3,000 ×, scale bar: 10 μm). **(B)** Part of the capillary bile duct is dilated; a large number of microvillous and lysosomes are visible (9,000 ×, scale bar: 5 μm). **(C)** Hyperplasia of collagen fibers can be seen in liver cells, with cross-sections (*). The picture also presents with an increased number of mitochondria with abnormal morphology (⋆) and an increased density of rough endoplasmic reticulum (▴) (9,000 ×, scale bar: 5 μm).

To further confirm the liver damage, electron microscopy of the liver biopsy was performed. As shown in Figure 2, hepatocytes were irregular in shape and size with an eccentric nucleus (Figure 2A, 3,000 ×, scale bar: 10 μm), capillary bile ducts were dilated, and a large number of microvilli and lysosomes were visible in hepatocytes (Figure 2B, 9,000 ×, scale bar: 5 μm). In addition, an increased number of mitochondria with abnormal morphology and an increased density of rough endoplasmic reticulum (ER) were observed (Figure 2C, 9,000 ×, scale bar: 5 μm). The patient's clinical symptoms were improved soon by managing with supportive treatment, including renal replacement therapy (CRRT), thyroid hormone supplementation, intravenous antibiotics, and glucose infusion (Table 1). Finally, the patient was discharged with full recovery after 1 month of hospitalization. During the recent 9-month follow-up period, she had complete clinical and biochemical recovery and did not require any additional treatment.

**Table 1:** Laboratory findings at clinical presentation and during hospitalization.

**Parameter (normal value)**	**D1**	**D4**	**D5**	**D8**	**D12**	**D17**	**D27**	**D28**
TB (3.4–17.1 μmol/L)	35.81	38.91	25.76	9.66	8.04	11.08	7.24	12.2
DB (0–6.8 μmol/L)	24.5	35.55	21.7	7.74	6.24	3.1	4.59	5.7
ALT (5–40 U/L)	7,169	1,189	772	381	176	91	29	25
AST (8–40 U/L)	9,677	238	93	30	37	35	30	29
γ-GGT (7–32 U/L)	40	97	86	113	100	120	65	61
Albumin (38–54 g/L)	22	15	14	19	18	27	22	21
Blood ammonia (11–51 μmol/L)	85	47	63	51	17	39		
PT (9.8–12.1 s)	23.5	15	12.4	12.9	12.0	10.7	11.8	11.4
INR (0.82–1.25)	2.1	1.32	1.08	1.13	1.04	0.93	1.02	0.98
Cre (18–66 μmol/L)	179	524	240	324	168	79	49	45
T3 (1.34–3.70 nmol/L)			0.81					2.3
T4 (64.30–158.70 nmol/L)			54.4					109.1
FT3 (3.58–6.92 pmol/L)			2.84					6.3
FT4 (9.60–14.50 pmol/L)			9.35					11.5
TSH (0.90–4.00 uIU/mL)			0.1					2.39

*D, day; DB, direct bilirubin; TB, total bilirubin; ALT, alanine transaminase; AST, aspartate transaminase; γ-GGT, γ-glutamyl-transferase; PT, prothrombin time; INR, international normalized ratio; Cre, serum creatinine; T3, triiodothyronine; T4, thyroxine; FT3, free triiodothyronine; FT4, free thyroxine; TSH, thyroid-stimulating hormone*.

## Discussion

In this report, we presented a Chinese pediatric RALF case with a novel compound heterozygote mutation, c.3596G> A (p.C1199Y)/ex.9del (p.216-248del), in the *NBAS* gene. In addition to the liver phenotype, she also had acute kidney injury, hypertension, mild pulmonary hypertension, pleural effusion, and hypothyroidism. Other extrahepatic manifestations, including skeletal involvement, growth failure, intellectual disability, ophthalmic abnormalities, facial dysmorphism, and cardiac abnormalities, were not observed. The c.3596G> A (p. C1199Y) mutation has been previously reported together with other mutation sites of the *NBAS* gene as a cause of RALF and was found to be a unique mutation among Chinese patients (5–7). A recent study (3, 7) reported that nine Chinese pediatric patients carried the c.3596G> A (p. C1199Y) mutation. The c.3596G>A (p. C1199Y) mutation is located in the strictly conserved Sec39 domain of NBAS and can lead to structural changes throughout the Sec39 region or the entire protein (5). The ex.9 del (p.216-248del) variant is located in the β-propeller and is predicted to be deleterious *in silico*, which was first reported in this study. Phenotype–genotype correlation studies revealed that pediatric patients with NBAS deficiency caused by mutations in the Sec39 domain have a predominant liver phenotype (infantile liver failure syndrome type 2/ILFS2, OMIM 616483), and variants in the β-propeller usually showed liver damage combined with multiorgan/system abnormalities (3, 7). Our patient presented a predominant liver phenotype, which was consistent with reported NBAS deficiencies caused by mutations located in the Sec39 domain (3, 7). However, compared with other reported cases with mutations in the β-propeller, such as c.680-690dupACTGTTTCAGC/p. Phe231ThrfsTer35, c.686dup/p. Ser230Glnfs^*^4, and c.680A > C/p. His227Pro (7–9), this patient had no clinical manifestation resembling SOPH syndrome. Interestingly, our patient had symptoms of hypertension, pleural effusion, and pulmonary hypertension, which further expanded the clinical spectrum of *NBAS* deficiency.

Currently, the exact mechanism of *NBAS* mutations causing RALF is not fully understood. The NBAS protein is a component of a soluble N-ethylmaleimide-sensitive factor attachment protein receptor (SNARE) complex, the syntaxin 18 complex, which is involved in Golgi-to-ER transport (10, 11). The NBAS protein interacts with p31 and ZW10-RINT-1, and it was shown that NBAS depletion reduced p31 expression and caused redistribution of Golgi recycling proteins accompanied by a defect in protein glycosylation (2, 10). p31 protein defects can accelerate hepatic lipogenesis and induce hepatocyte apoptosis via increasing the relative expression of stress response genes in the ER (12). The syntaxin 18 complex is thermally susceptible, as evidenced by an increased sensitivity to high temperature at the protein and functional levels and a disturbed tethering of vesicles in the skin fibroblasts of patients with NBAS mutations (2). The catabolic state and high energy consumption during fever further impair the function of the syntaxin 18 complex, resulting in ER stress (13). ER stress triggers liver cell destruction by accelerating lipogenesis and activating the unfolded protein response (14). Thus, mechanisms of fever-dependent ALF in NBAS deficiency are proposed to be associated with altered Golgi–ER retrograde transport and ER stress. In addition, NBAS plays a pivotal role in non-sense-mediated mRNA decay (NMD), a conserved posttranscriptional RNA surveillance pathway (15). NMD recognizes and degrades mRNAs bearing premature termination codons (PTCs) to avoid the accumulation of truncated proteins and their cytotoxicity (16). The NMD pathway is important for liver development, function, and regeneration (17). NBAS was found to coregulate target genes with core NMD factors and participate in a negative feedback regulatory loop in the NMD pathway (18). It was shown that NBAS-mediated NMD modulates genes involved in bone development and cholesterol biosynthesis, which may explain the skeletal dysplasia and Pelger–Huët anomaly in SOPH syndrome (18).

Given the rarity of RALF being caused by *NBAS* mutations, other genetic causes of RALF in children should be considered before diagnosis. Studies have revealed that deleterious mutations in cytosolic leucine-tRNA synthetase (LARS) and RINT1 gene mutations are common in liver failure syndrome-1 (ILFS1) (19) and liver failure syndrome-3 (ILFS3) (20), respectively. ILFS1 is characterized by the impairment of liver synthetic function, including hypoalbuminemia and severe coagulopathy, with mild or without hepatocellular injury or defects in liver detoxification during the early phase of the disease (19). The main clinical manifestations of ILFS3 are similar to ALF caused by *NBAS* mutations, presented with fever-related RALF, vomiting, hypoglycemia, coagulopathy, hyperammonemia, and skeletal abnormalities (20). However, there are differences in skeletal phenotypes: vertebral body abnormalities, including anterior breaking and irregularity, are common in ILFS3 (20), while NBAS deficiency has been characterized as atypical osteogenesis imperfecta with bone fragility (9). Furthermore, Wolcott–Rallison syndrome (WRS, OMIM 226980) (21) and CALFAN syndrome (OMIM 616719) (22, 23) should also be taken into consideration. WRS is caused by mutations in the gene encoding eukaryotic translation initiation factor 2α kinase 3 (EIF2AK3) and is characterized by neonatal/early-onset non-autoimmune insulin-requiring diabetes associated with skeletal dysplasia, RALF, renal dysfunction, exocrine pancreas insufficiency, intellectual deficit, hypothyroidism, neutropenia, and recurrent infections (21). Permanent early-onset diabetes is a typical manifestation of WRS (21). The clinical presentations of CALFAN syndrome included recurrent low γ-GGT cholestasis or ALF with onset in infancy and a variable neurological phenotype of later onset, which was caused by SCYL1 mutations (22, 23). However, the symptoms of the nervous system are less common in NBAS deficiency (9). Although those diseases involved with ALF showed similar clinical manifestations with multisystem involvement, genetic testing is valuable to help clinicians differentiate them. In addition, it needs to be distinguished from additional genetic diseases that cause ALF, such as PCK1 and PCK2 (24). In this reported case, only NBAS mutations were observed by WES, which ruled out other genetic causes of ALF.

There is no known cure treatment for patients with NBAS deficiency, and we can individualize supportive treatment and emphasize the significance of follow-up investigations. More proactive infection prevention strategies are required to avoid ALF episodes during the interval. Early and effective antipyretic therapy is necessary to prevent the later occurrence of ALF. The application of intravenous glucose and lipids can alleviate disease severity and promote recovery, as reported in the literature (9). Patients with hepatic encephalopathy (HE) or decreased serum carnitine levels can receive L-carnitine supplementation (25). ALF can be lethal in patients without treatment. Liver transplantation is the only rescue therapy for patients with end-stage ALF. Thus, early diagnosis is critical for the treatment and management of pediatric ALF.

In summary, we reported that a novel compound heterozygote mutation in the *NBAS* gene caused fever-related RALF in a Chinese child, which further expands the mutational spectrum of *NBAS*. Genetic testing by NGS is valuable in the differential diagnosis of pediatric hereditary liver diseases.

## Data Availability Statement

The original contributions presented in the study are included in the article/supplementary materials, further inquiries can be directed to the corresponding author/s.

## Ethics Statement

Written informed consent was obtained from the individual(s), and minor(s)' legal guardian/next of kin, for the publication of any potentially identifiable images or data included in this article.

## Author Contributions

TZ and YW conceived the study and edited the manuscript. BJ and YW drafted the manuscript. FX, XL, YX, and YW acquired, analyzed, and interpreted the data. All authors agreed to be accountable for all aspects of the work.

## Conflict of Interest

The authors declare that the research was conducted in the absence of any commercial or financial relationships that could be construed as a potential conflict of interest.
